# Physical Activity and its Associations with other Lifestyle Elements in Polish Women

**DOI:** 10.2478/v10078-011-0050-9

**Published:** 2011-10-04

**Authors:** Maria Alicja Nowak

**Affiliations:** 1 University School of Physical Education in Poznań, Branch Faculty of Physical Culture in Gorzów Wielkopolski, Poland

**Keywords:** physical activity, women, lifestyle, health behaviors

## Abstract

The aim of the study was to determine associations between physical activity and other elements of women’s lifestyle (nutrition, being a nonsmoker, moderate alcohol consumption, medical check-ups).

Between 1999 and 2004, 1361 women aged 20–75 were studied. They were inhabitants of cities located in the west of Poland and engaged in physical activity (purposeful selection). The subjects fell into four groups depending on the length of their physical exercise history: G I – those who had been exercising for 1 year; G II [1–4); G III [4–6); G IV [≥7). The diagnostic poll method was employed, (questionnaire and interview techniques). For the verification of the research hypotheses concerning the influence of socio-demographic factors on women’s physical activity, the evaluation of changes in health-related behaviors resulting from long term physical activity, indication of associated behaviors, the independence χ^2^ test and multiple correspondence analysis were used.

Women’s physical activity was found to be related to maintenance of proper weight (BMI) (p≤0.05), moderate consumption of low-alcohol beverages (p≤0.05) and regular dental check-ups (p≤0.05). Despite more frequent attempts to take up smoking, the respondents gave up the habit two times as often as the whole population of women in Poland. These correlations were more apparent among women with longer exercise histories, who mostly had post-secondary education. Occurrence of associated behaviors affecting health positively and negatively was also shown, the latter concerning a smaller group of respondents.

The noticed correlations between physical activity and health behaviors, which comprehensively influence lifestyle, may be of importance in gradual reduction of risk factors.

## Introduction

There might be little influence of physical exercise on reducing separate risk factors, but the cumulative effects, through comprehensive influence on human organism, may be significant for health (Åstrand, 1997). Factors connected with lifestyle (including smoking, consumption of alcoholic beverages, low physical activity, obesity) are the most serious source of health threats (World Health Report, 2002). Association of occurrences of health hindering factors increases the threat of disease. Research has confirmed correlations between mortality and anti-health lifestyle, including low physical activity (Bauman, 2004; Van Der Wilk and Jansen, 2005). Research conducted in the USA, Australia and several European countries has shown a low level of physical activity to be linked to overweight and obesity (Pomerlau et al., 1997; Salmon et al., 2000). Physical activity becomes even more important with advancing age, as it may improve cognition and the ability to engage in activities of daily living (given its critical role in balance and mobility as well as bone strength) (Warburton et al., 2006). Previous research in middle-aged adults has highlighted the effectiveness of physical activity in preventing weight gain over time (Litman et al., 2005). Sixty-five-plus-year-olds’ participation in modest amounts of physical activity attenuated age-related weight loss by approximately 25% with little additional benefit observed at higher physical activity levels (Stephen and Janssen, 2010).

Associations between engaging in physical activity and smoking cigarettes are ambiguous (Dishman and Sallis, 1994). Relationships between recreational physical activity of women aged 18–74 and their attitudes toward smoking were not found (Chung et al., 2007). Weak correlations between these behaviors observed in a cross-sectional study into healthy aging was put down to the fact that smokers included in the research had not reached 60 years of age (Peel et al., 2005). Whereas a low level of physical activity, associated with tobacco consumption, obesity and overweight, was characteristic of subjects with the lowest education and income (Wardle and Steptoe, 2003).

According to one study, there is a weak correlation between drinking alcoholic beverages and physical activity (Wankel and Sefton, 1994; Johnson et al., 1998; Chung et al., 2007); according to another study, engaging in physical activity in spare time is linked to moderate alcohol consumption (Smothers and Bertolucci, 2001). Moderate consumption of alcoholic beverages is not a threat to health, but a factor preventing occurrence of cardiovascular disease (Wannametheee, 1999). Therefore, official healthy lifestyle recommendations do not mention unconditional abstinence from, but rather moderate consumption of such beverages.

The choice of health-related behaviors is of great importance for leading a healthy lifestyle. The relation between risk perception and behaviors as lifestyle elements, however, is rather remote and subject to various conditions, such as health awareness (Wardle and Steptoe, 2003). We can talk about lifestyle when we deal with certain configurations of behavior patterns (relatively constant and repetitive) in situations where there are conditions for alternative behaviors. This is the case with women engaging in physical activity over a longer period of time. Factors influencing many-year physical activity in women of different ages are less known. Available research results concern rather benefits of and barriers preventing from physical activity, perceived subjectively or existing objectively (Azevedo, 2007), and regarding people with short histories of recreational physical activity. No results of research into Polish women participating in recreational activities over many years, i.e. leading an active lifestyle, have been traced. Research results have not been analyzed in relation to the length of their physical activity.

The aim of the study was to determine associations between physical activity and other elements of women’s lifestyle (nutrition, being a nonsmoker, moderate alcohol consumption, medical check-ups).

The following hypotheses were assumed:

1. Women’s physical activity is affected by age, education and employment.
2. In women who are physically active for many years, changes to their lifestyles occur (nutrition, tobacco and alcohol consumption, medical check-ups).
3. Long term physical activity is associated with health behaviors (women’s proper body mass, giving up smoking, moderate consumption of alcoholic beverages and regular dental check-ups).

## Material and Methods

### Subjects

Research into women’s lifestyle has been conducted by the author and trained interviewers since 1995. The study of women who had been participating in recreational activities for at least a year included 1361 subjects aged 20–75, living in cities of western Poland. The results were gathered between 1999 and 2004. The use of purposeful sampling was a significant limitation in carrying out the study (according to research conducted by Kearney et al. (1999) about 50% of subjects gave up exercising after 3–6 months), but it made it possible to analyze associations between the length of physical activity history and chosen health behaviors. The length of the women’s physical activity was expressed as the number of years of taking exercise, determined in interviews with the subjects and verified by the physical recreation instructors (Vuillemin et al., 2000; Shepard, 2002). On grounds of the results obtained individually for each woman, the lower quartile (25th percentile), the median (50th percentile) and the upper quartile (75 percentile) were determined and thus four groups were formed (Table 1).

The majority of the subjects participated in organized exercise twice a week (49.6%) or more often (36.9%), devoting to it 61 to 120 minutes (47.8%) or more (41.7%).

The prevailing organized form of recreational physical activity was gymnastics in its contemporary and traditional varieties (about 80% of the responses). Further places were taken by swimming (about 10%) and individually practiced jogging and cycling (just over 10%). Seasonal sports, sports games and martial arts were practiced by a total of 4% of the examined.

### Data collection and procedures

In order to analyze physically active women’s lifestyles the diagnostic poll method was employed, with the use of the techniques of questionnaire and interview, health behavior scale and document analysis. Prior to commencing the objective study, the questionnaire technique was checked in the pilot study conducted in 1997 by the author and trained pollsters. It included women of different ages, inhabitants of cities and villages, employees of chosen institutions, students, parents of elementary, secondary and higher education schools students, physically active and passive individuals (over 2.5 thousand subjects in total). An analysis of the study results enabled the author to verify the questionnaire (modification of closed, half open, multiple choice and single choice cafeteria-style questions) and confirm the appropriateness of the tool used. A high reliability of the tool was also confirmed, through repeated examination of chosen subjects (about 30) a month afterwards. Partial results of the pilot study were published. The survey questionnaire was additionally verified by the results of the Attitudes Scale Pro-Zet (created according to the Likert scale), with confirmed accuracy and reliability. Eventually the research area was limited to women living in cities, systematically participating in physical exercise.

In the present article, the material from the questionnaires and interviews was used, directly concerning the subjects’ socio-demographic characteristics (age, education and employment) and their health-related behaviors, such as physical activity, nutrition, tobacco and alcohol consumption and attending regular medical check-ups. The categories with respect to the women’s careers were: working, permanently passive (retirees or ill-health pensioners), temporarily passive (being on child-raising leave, unemployed) and learning or studying (except for physical education students). On grounds of the information about current body mass and height reported by the respondents, the BMI was calculated. According to the most often assumed criteria (Howley and Franks, 1997; The European Health Report 2002), the proper BMI for women should vary from 20.0 to 24.9 kg/m^2^; values ranging from 25.0 to 29.9 kg/m^2^ indicate overweight, equal to or exceeding 30.0 kg/m^2^ – obesity, and below 20.0 kg/m^2^ – underweight. As far as smoking cigarettes is concerned, among the physically active women were those who: did not smoke, had been smoking regularly (at least 1 cigarette a day for half a year), had been smoking occasionally, had given up smoking. In the evaluation of alcoholic beverages consumption 5 categories were distinguished with respect to frequency and beverage type: not drinking, drinking low-alcohol beverages (rarely – 1–2 times a month or less often; often – 1–2 times a month or more often) and drinking high-alcohol beverages (rarely; often). Health control was evaluated on grounds of the frequency of dental check-ups (in the past 6 months, 6–12 months before, 1–2 years before, earlier) as well as gynecological ones (in the past year, over a year but not earlier than 2 years before, earlier than 2 years before, never).

### Statistical Analysis

For the verification of the research hypotheses concerning the influence of age, education and employment on women’s physical activity, as well as for the evaluation of changes in health-related behaviors resulting from physical activity and the indication of associated behaviors, frequency of features and the independence χ^2^ test were used. In search of correlations between physical activity determinants and the choice of health behaviors, multidimensional correspondence analysis was employed. The method makes it possible to illustrate graphically and comprehensively the associations between data, which are qualitative for the most part (Van Buuren and de Leeuw, 1992). The variables with their categories are presented on the plane. Closeness of particular categories indicates a more direct relation between them. In connection with the results from the χ^2^ test it facilitates analysis of all the variables and their categories which are significant in determining the examined associations. Statistica 8.0 software package (StatSoft, Inc. USA) was used to make calculations.

## Results

It was found that participation in exercise was characteristic of women under 50 years of age (72.5%). Those who were over 50 and exercised, had been physically active for a longer period of time (G III and G IV) (p≤0.001). Subjects with a secondary and pre-secondary education had shorter physical activity histories **(G I and G II),** whereas those with higher education – longer (G III and G IV) (p≤0.001). The respondents were mostly employed. The women who were permanently passive as far as their careers are concerned, more often fell in G III and G IV (p≤0.001) (Table 2).

The correlations between the length of physical activity and health behaviors are presented in Table 3. A statistically significant dependence was found between the length of physical activity and the values of the BMI (p≤0.05). The longer the physical activity history the greater number of respondents had proper body mass (BMI of [20.0–25.0 kg/m^2^) (G I – 51.4%; G IV – 61.1%). Overweight as well as obesity were more often characteristic of women with shorter physical activity history; in both cases subjects from G I (23.2% and 6.5% resp.).

Statistically significant dependences – between the length of the subjects’ physical activity and their attitudes toward tobacco consumption were not found. Smoking cigarettes (regularly or irregularly) was still reported by 13.2% of the women. Out of 41.6% of those who had ever smoked, 28.4% had given up the habit.

The respondents who declared consumption of alcoholic beverages, mainly drank low-alcohol ones (beer, tinctures, liqueurs), 1–2 times a month or less often (rarely) (62.5%) (p≤0.05). Consumption of high-alcohol beverages 1–2 times a month or less often (rarely) was reported by more women from G I and G II (11.9% and 9.2% respectively), although respondents from G II also drank high-alcohol beverages often (1–2 times a week or more often) (5.5%).

Most of the exercising women displayed proper behavior as far as dental check-ups are concerned (60.5%); they slightly more often belonged to G III and G IV. Check-ups carried out 1–2 years before were mainly characteristic of respondents from G I (15.7%) and G II (13.0%) (p≤0.05).

No statistically significant correlations with regard to the frequency of undergoing gynecological check-ups by women from different groups were found. Most respondents had also gone to gynecological check-ups in the past year (64.6%), but there were 16.7% of the women who had not had these check-ups for over 2 years.

Smoking cigarettes was linked to alcoholic beverages consumption (Table 4). Women who had never smoked and those who had given up smoking were more often in the not drinking group (15.3% and 9.6% respectively) (p≤0.001). Women who smoked regularly and irregularly were characterized by high-alcohol beverages consumption 1–2 times a month or less often (13.8% and 22.0% respectively) and 1–2 times a week ore more often (5.1% and 4.9% resp.). Irregular smokers and women who had given up the habit had similar behaviors concerning the choice of low-alcohol beverages and the frequency of their consumption (1–2 times a month, 1–2 times a week). At the same time, irregular smokers preferred high-alcohol beverages more often than the others (22%). A statistically significant dependence was observed between the frequency of the subjects’ dental and gynecological appointments (p≤0.001) (Table 5).

Out of 873 women who had undergone gynecological examination in the past year, 581 (66.6%) had seen the dentist in the past 6 months. It could be seen as an association of positive **behaviors.** Association of negative behaviors occurred to a much lesser degree. Out of 224 women who had undergone gynecological examination 2 years before or earlier, 20 (8.9%) had not had a dental appointment for over 2 years as well.

**Figure 1:**
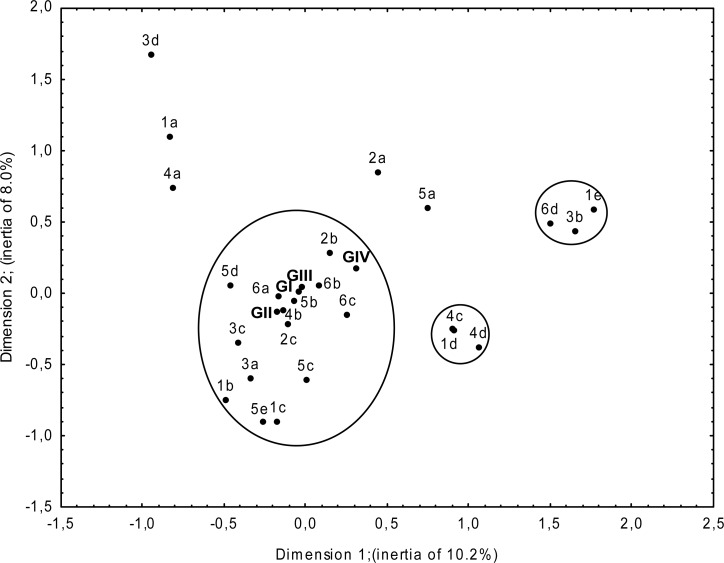
Relationships between women’s physical activity and BMI, alcoholic beverages consumption, undergoing dental check-ups, age, education and employment situation

In order to comprehensively present chosen correlations, multidimensional correspondence analysis was used (Table 6, Fig. 1). It was found that: women engaging in regular physical activity (G I, G II, G III, G IV), working at jobs (3a) or temporarily not working (3c), were aged 30–39 (1b) and 40–49 (1c), and had secondary (2b) or post-secondary education (2c).

These women had more often proper body mass (BMI of [20.0–25.0 kg/m^2^) (4b). They were characterized by drinking low-alcohol (5b) and high-alcohol beverages (5c) with low frequency (1–2 times a month and less often), high-alcohol beverages being consumed more frequently (5e) mainly by respondents from G II. Women from G I – G III had mostly had dental check-ups in the past 6 months (6a) and in the past 6–12 months (6b), whereas among those who had done it 1–2 years before (6c) there were more subjects from G IV with older respondents.

A peripheral position was occupied by women aged 50–59 (1d), overweight (4c) or obese (4d), and women aged 60–75 (1e), permanently passive in their careers (3b), who had undergone dental examination over 2 years before (6d).

Studying or learning women (3d), aged 20–29 (1a) were slightly more often underweight (4a). Subjects with a pre-secondary education (2a) as well as those who did not drink alcoholic beverages constituted a minority.

## Discussion

The study results confirm previous findings, that participation in physical recreation is characteristic of better educated women (Kearney et al., 1999), having good material and employment situations, mainly living in cities (Rütten et al., 2001). Increased engagement in exercise among women over 60 years of age has also been found (McTiernan et al., 1998). A rise in the number of women over 60 taking up exercise, especially among subjects with the longest exercise history, was confirmed in the present study. This situation can be explained by the increased amount of free time during retirement and educated women’s ability to manage it.

A great deal of research has shown a positive relationship between physical activity and maintaining proper body mass, and preventing overweight and obesity (Jakicic et al., 2002; Wenche et al., 2004). American female students who engaged in physical activity paid more attention to the quality of their diet and were characterized by lower consumption of fatty foods (Johnson et al., 1998). 36% of subjects who had been physically active for 6 months or longer had proper BMI. Among EU inhabitants engaging in physical exercise 31% were underweight, 26% overweight and 16% obese (Kearney et al., 1999). The findings of the present study permit the observation that physically active women - in comparison with a representative sample of female inhabitants of other cities in Poland aged 20–64, about 45% of whom did not engage in physical recreation in their free time - much more often have BMI <25.0 kg/m^2^ (75.7% and 48.2% resp.), are less often overweight (20.4% and 30.3% resp.) and many times less often obese (3.9% and 21.6% resp.) (Kwaśniewska et al., 2007). This dependence is also evident in comparison with those who had been exercising for six months or longer (Kearney et al., 1999). Whereas in a long term analysis of some associations of physical activity (job-related and recreational), it was noticed that obesity was less common among physically active people compared with those leading a sedentary lifestyle (Anderssen et al., 2008).

A small number of obese women probably does not result solely from taking physical exercise. Obese women are reluctant to undertake physical effort. Research into obese adults’ participation in physical activity showed that exercising in order to lose body mass was the least popular (Prevalence of leisure-time physical activity among overweight adults – United States 1998Prevalence of leisure-time physical activity among overweight adults – United States 2000). Numerous cultural barriers intensify obese people’s reluctance to exercise (Loland, 1998). Apparently obese people less often take up exercise and keep it up for several years. This group, increasing steadily in many societies, requires special attention in creating favorable conditions for physical activity.

Tobacco smoking is relatively widespread in Polish population, as it is in the countries of Central and Eastern Europe (Molarius et al., 2001). Statistically significant dependences between the length of the subjects’ physical activity and their attitudes toward smoking were not found. However, physically active females, in comparison with the population of women in Poland (Central Statistical Office, 2007), despite more frequent attempts to take up smoking (41.6% and 36.4% resp.), gave up the habit two times as often (28.4% and 14.2% resp.). The smoking habit in Polish population concerns 23.1% of women and 13.2% of the subjects.

Positive relationships between exercising and avoiding cigarettes were found among students. Those who had exercised several times in the past two weeks more often belonged to the non-smoking group (Steptoe et al., 1997). A study of women aged 50–64 has shown that the percentage of smokers is lower among the physically active ones (the dependence was constant), whereas a sedentary lifestyle is conducive to tobacco consumption (McTiernan et al., 1998). Many-year research has indicated that healthy aging is favored by being a non-smoker for the whole life or for at least 15 years, as well as by having a high level of physical activity (Haveman-Nies et al., 2003). Combining positive health behaviors of high physical activity with not smoking enhanced the chances of healthy aging (Leveille et al., 1999). A high proportion of women who had given up smoking may serve as evidence of the awareness of smoking-related threats and the effectiveness of the preventive programs conducted in recent years, as well as the legal regulations, e.g. ban on smoking in public places.

A comparison drawn between the subjects’ attitudes toward alcoholic beverages consumption and nationwide data (Central Statistical Office, 2007) permits the observation that among physically active women there is a lower percentage of those who do not drink alcohol (12.3% and 32.3% resp.). Increased abstinence because of participation in physical exercise was not observed. Similar findings have been reported (Westersterp et al., 2004). Physically active women were mainly characterized by low-alcohol beverages consumption 1–2 times a month or less often. Leisure-time physical activity and a moderate weekly alcohol intake are both important to lower the risk of fatal ischaemic heart disease and all-cause mortality (Pedersen et al., 2008).

According to some research findings, physical activity is associated with non-smoking, alcohol abstinence or consumption in moderate amounts and with moderate frequency (Mensink et al., 1997; Smothers and Bertolucci, 2001; Aarnio et al., 2002; Jakicic et al., 2002). These relationships were confirmed in the present study. Subjects who had never smoked and those who had given up the habit were more often non-drinking. At the same time, there is an interdependence between alcoholic beverages consumption and smoking cigarettes. Women who smoked regularly and irregularly were characterized by high-alcohol beverages consumption, with various frequency. Association of these two anti-health behaviors is probably customary in character.

A statistically significant dependence was found between the frequency of subjects’ dental and gynecological appointments. Most women who had undergone gynecological examinations in the past year had also seen the dentist in the last 6 months. It could be seen as an association of positive behaviors. Association of negative behaviors occurred to a much lesser degree. The number of women displaying proper behaviors concerning dental examinations increased with the length of physical activity history.

## Conclusions

The study revealed associations between women’s physical activity and other lifestyle elements, such as maintaining proper body mass, moderate low-alcoholic beverages consumption, regular dental check-ups. Despite more frequent attempts to take up smoking, the subjects gave up the habit two times as often as the whole population of women in Poland. These correlations were more apparent among women with longer exercise histories, who mostly had post-secondary education.

Occurrence of associated behaviors affecting health positively and negatively was also shown, the latter concerning a smaller group of respondents (smoking and high-alcohol beverages consumption; avoiding gynecological and dental examinations).

The associations found between physical activity and health-related behaviors do not mean automatic elimination of health-threatening behaviors among physically active women. However, through comprehensive influence on lifestyle, they may be of significance in gradual reduction of risk factors.

Health threats can be decreased through intensive promotion of activities in schools, health care centers, workplaces, by creating greater personal responsibility for one’s own health, keeping in mind the possibility of shaping a pro-health lifestyle, taking into account the existing systemic limitations.

## Figures and Tables

**Table 1:** Division of subjects into groups depending on the length of physical activity history

Division into groups	Percentile [C]	Number of years of exercise	Group size	
			*n*	%
Group I	(0, C25]	1 year	397	29.2
Group II	(C25, C50]	[1; 4); (over 1 year, but below 4)	380	27.9
Group III	(C50, C75]	[4; 7); (4 or more years, but below 7)	252	18.5
Group IV	(C75,100]	≥7; (7 or more years)	332	24.4
		Total	1361	100

**Table 2:** Dependence between sociodemographic factors and the length of physical activity

Sociodemographic variables	Length of physical activity				Total		*p* for χ^2^ test

	G I	G II	G III	G IV			

	%				*n*	%	
Age (in years)							
20–29	32.7	27.6	28.6	23.8	386	28.4	
30–39	23.7	26.6	17.0	12.6	280	20.6	
40–49	21.7	24.7	25.8	22.6	320	23.5	***
50–59	10.6	10.8	13.1	16.0	169	12.4	
60–75	11.3	10.3	15.5	25.0	206	15.1	

Education							
Pre-secondary	4.5	5.3	3.6	3.3	58	4.3	
Secondary	41.3	35.8	27.9	28.3	464	34.1	***
Post-secondary	54.2	59.0	68.5	68.4	838	61.6	

Employment							
Working	59.4	55.7	55.0	50.0	751	55.3	
Permanently passive	18.1	17.7	23.1	29.8	296	21.8	**
Temporarily passive	9.1	8.2	6.0	6.0	102	7.5	
Learning and studying	13.4	18.5	15.9	14.2	210	15.4	

** *- significantly different p*≤*0.01,*

*** *- significantly different p*≤*0.001*

**Table 3:** Dependences between the length of physical activity and BMI, alcoholic beverages consumption, tobacco consumption and undergoing dental and gynecological check-ups

Health behaviors	Length of physical activity				Total		*p* for χ^2^ test

	G I	G II	G III	G IV			

	%				*n*	%	
Value of BMI [kg/m^2^] (n=1361)							
< 20.0	18.9	17.4	19.4	18.1	250	18.4	
[20.0–25.0)	51.4	58.7	59.5	61.1	780	57.3	*
[25.0–30.0)	23.2	20.8	19.1	17.8	278	20.4	
≥30.0	6.5	3.1	2.0	3.0	53	3.9	

Tobacco consumption (n=1355)							
Have never smoked	56.9	57.5	61.9	58.4	791	58.4	
Regularly smoking	11.7	11.1	7.5	9.4	138	10.2	n.s.
Irregularly smoking	3.3	2.9	2.8	3.0	41	3.0	
Have given up (ex-smokers)	28.1	28.5	27.8	29.2	385	28.4	

Frequency and kinds of consumed alcoholic beverages (n=1359)							
Not drinking	11.8	10.0	13.1	14.8	167	12.3	
Rarely consuming low-alcohol beverages	64.2	63.2	63.4	59.0	849	62.5	
Rarely consuming high-alcohol beverages	11.9	9.2	5.2	8.4	123	9.0	*
Often consuming low-alcohol beverages	9.3	12.1	14.7	15.1	170	12.5	
Often consuming high-alcohol beverages	2.8	5.5	3.6	2.7	50	3.7	

Frequency of dental check-ups (n=1355)							
-In the last 6 months	57.7	59.3	63.1	63.3	820	60.5	
-6–12 months ago	23.3	23.5	24.6	26.1	329	24.3	*
-1–2 years ago	15.7	13.0	9.1	7.0	157	11.6	
- Earlier	3.3	4.2	3.2	3.6	49	3.6	

Frequency of gynecological check-ups (n=1351)							
-In the last year	59.4	64.8	70.5	66.1	873	64.6	
-Over a year but not earlier than 2 years ago	22.7	17.2	14.3	19.1	253	18.7	n.s.
-Earlier than 2 years ago	17.9	18.0	15.1	14.9	225	16.7	

** *- significantly different p*≤*0.05. n.s. - no statistical significance*

**Table 4:** Associations between tobacco consumption and alcoholic beverages consumption in physically active women

	Tobacco consumption				Total	
Alcoholic beverages consumption	Regularly smoking (n=138)	Have never smoked (n=791)	Have given up (n=386)	Irregularly smoking (n=41)		

	%				*n*	%
-Not drinking	4.4	15.3	9.6	2.4	165	12.2
-Rarely consuming low-alcohol beverages	64.5	64.2	58.8	58.5	848	62.5
-Rarely consuming high-alcohol beverages	13.8	6.7	10.9	22.0	123	9.1
-Often consuming low-alcohol beverages	12.3	10.6	16.6	12.2	170	12.5
-Often consuming high-alcohol beverages	5.1	3.2	4.1	4.9	41	3.7

*** *- significantly different p*≤*0.001*

**Table 5:** Associations between undergoing dental and gynecological check-ups

Dental check-ups	Gynecological check-ups			Total (n=1349)	

	In the last year (n=872)	1–2 years ago (n=253)	Earlier (n=224)		

	%			*n*	%
In the last 6 months	66.6	49.4	49.6	817	60.6
6–12 months ago	23.1	29.3	23.2	327	24.2
1–2 years ago	8.1	17.4	18.3	156	11.6
Earlier	2.2	3.9	8.9	49	3.6

*** *- significantly different p*≤*0.001*

**Table 6:** Relationships between women’s physical activity and BMI, alcoholic beverages consumption, undergoing dental check-ups, age, education and employment situation (explanations for figure 1)

Arrangement of column and row coordinates for n=1353							
**Explanation**	**Symbol**	**Dimension 1**	Dimension 2	**Explanation**	**Symbol**	Dimension 1	**Dimension 2**
Studying	3d	−0.951	1.674	Age: 40–49	1c	−0.174	−0.907
Age: 20–29	1a	−0.836	1.100	Often consuming high-alcohol beverages	5e	−0.263	−0.898
BMI: <20kg/m2	4a	−0.815	0.744	Age: 30–39	1b	−0.492	−0.755
Age: 30–39	1b	−0.492	−0.755	Rarely consuming high-alcohol beverages	5c	0.002	−0.609
Often consuming low-alcohol beverages	5d	−0.463	0.053	Employment: working	3a	−0.334	−0.596
Employment: temporarily passive	3c	−0.418	−0.344	BMI: >30kg/m2	4d 1.061		−0.375
Employment: working	3a	−0.334	−0.596	Employment: temporarily passive	3c	−0.418	−0.344
Often consuming high-alcohol beverages	5e	−0.263	−0.898	BMI: [25.0–30.0 kg/m2)	4c	0.912	−0.261
Group II	GII	−0.178	−0.127	Age: 50–59	1d	0.899	−0.251
Age: 40–49	1c	−0.174	−0.907	Education: post-secondary	2c	−0.112	−0.213
Dental check-up in the last 6 months	6a	−0.171	−0.021	Dental check-up 1–2 years ago	6c	0.254	−0.150
BMI: [20.0–25.0 kg/m2)	4b	−0.135	−0.120	Group II	GII	−0.178	−0.127
Education: post-secondary	2c	−0.112	−0.213	BMI: [20.0–25.0 kg/m2)	4b	−0.135	−0.120
Group I	GI	−0.075	−0.050	Group I	GI	−0.075	−0.050
Rarely consuming low-alcohol beverages	5b	−0.039	0.014	Dental check-up in the last 6 months	6a	−0.171	−0.021
Group III	GIII	−0.025	0.046	Rarely consuming low-alcohol beverages	5b	−0.039	0.014
Rarely consuming high-alcohol beverages	5c	0.002	−0.609	Group III	GIII	−0.025	0.046
Dental check-up 6–12 months ago	6b	0.081	0.052	Dental check-up 6–12 months ago	6b	0.081	0.052
Education: secondary	2b	0.149	0.282	Often consuming low-alcohol beverages	5d	−0.463	0.053
Dental check-up 1–2 years ago	6c	0.254	−0.150	Group IV	GIV	0.312	0.170
Group IV	GIV	0.312	0.170	Education: secondary	2b	0.149	0.282
Education: pre-secondary	2a	0.443	0.850	Employment: permanently passive	3b	1.656	0.440
Not consuming alcoholic beverages	5a	0.746	0.594	Dental check-up earlier than 2 years ago	6d	1.503	0.487
Age: 50–59	1d	0.899	−0.251	Age: 60–75	1e	1.767	0.588
BMI: [25.0–30.0 kg/m2)	4c	0.912	−0.261	Not consuming alcoholic beverages	5a	0.746	0.594
BMI: >30kg/m2	4d	1.061	−0.375	BMI: <20kg/m2	4a	−0.815	0.744
Dental check-up earlier than 2 years ago	6d	1.503	0.487	Education: pre-secondary	2a	0.443	0.850
Employment: permanently passive	3b	1.656	0.440	Age: 20–29	1a	−0.836	1.100
Age: 60–75	1e	1.767	0.588	Studying	3d	−0.951	1.674
